# Precision soft tissue balancing: grid-assisted pie-crusting in total knee arthroplasty

**DOI:** 10.3389/fsurg.2024.1331902

**Published:** 2024-04-05

**Authors:** Arash Rezaei, John Moon, Asher Lichtig, Barbara Mera, Brett Drake, Apurva S. Choubey, Sunjung Kim, Nicole Tueni, Hristo Piponov, Jason Koh, Joseph Karam, Farid Amirouche

**Affiliations:** ^1^ Department of Orthopaedics, University of Illinois at Chicago, Chicago, IL, United States; ^2^ University of Illinois College of Medicine, Chicago, IL, United States; ^3^ Department of Orthopaedic Surgery, Northshore University Health System, Skokie, IL, United States

**Keywords:** arthroplasty, pie-crusting, MCL, LCL, TKA, soft-tissue balancing

## Abstract

**Introduction:**

The varus and valgus knee deformities result from imbalance in tension between medial and lateral soft tissue compartments. These conditions need to be addressed during total knee arthroplasty (TKA). However, there is no consensus on optimal soft-tissue release techniques for correcting varus and valgus deformities during TKA. We assessed the efficacy of a novel grid-based pie-crusting technique on soft-tissue release.

**Methods:**

Cadaver knees were dissected, leaving only the femur and tibia connected by an isolated MCL or the femur and fibula connected by an isolated LCL. Bone cuts were made as performed during primary TKA. Mechanical testing was performed using an MTS machine. A 3D-printed 12-hole grid was placed directly over the MCL and LCL. Using an 18-gauge needle, horizontal in-out perforations were made 3 mm apart. Deformation and stiffness of the ligaments were collected after every 2 perforations. Means were calculated, and regression analyses were performed.

**Results:**

A total of 7 MCL and 6 LCL knees were included in our analysis. The mean medial femorotibial (MFT) space increased from 6.018 ± 1.4 mm–7.078 ± 1.414 mm (*R*^2 ^= 0.937) following 12 perforations. The mean MCL stiffness decreased from 32.15 N/mm–26.57 N/mm (*R*^2 ^= 0.965). For the LCL group, the mean gap between the femur and ﬁbula increased from 4.287 mm–4.550 mm following 8 perforations. The mean LCL stiffness decreased from 29.955 N/mm–25.851 N/mm. LCL stiffness displayed a strong inverse relationship with the number of holes performed (*R*^2 ^= 0.988).

**Discussion:**

Our results suggest that using this novel grid for pie-crusting of the MCL and LCL allows for gradual lengthening of the ligaments without sacrificing their structural integrity. Our proposed technique may serve as a valuable piece in the soft-tissue release toolkit for orthopaedic surgeons performing TKA in varus and valgus deformed knees.

## Introduction

Total Knee Arthroplasty (TKA) is an increasingly popular procedure, with yearly surgeries in the United States projected to be well over 1,000,000 in the next 10 years (1). Much of the success of a TKA is attributed to proper knee balancing during surgery (2). During trialing, proper knee balancing is deﬁned as a mediolateral load differential of less than 15 pounds (2). One of the first philosophies proposed to balance the knee during TKA was the mechanical alignment (MA) technique. A core tenet of this philosophy is that alignment can be restored through soft-tissue release and ligament balancing, rather than bone resection (3). This “balancing” is done intraoperatively through various surgical corrections, which include Iliotibial band (ITB) release, femur recut, tibia recut, posterolateral capsule (PLC) release, popliteus release, arcuate release, medial collateral ligament (MCL) pie-crusting, and alterations of insert thickness (2, 4). Adequate soft tissue balance in tension is essential to TKA surgery (1, 5–9). The tension on the medial and lateral sides must be equalized to have balanced ﬂexion and extension gaps (6). This balancing becomes more complicated when patients present with preoperative varus or valgus knee deformities, in which case the malformation cannot be corrected without appropriate soft tissue release (2).

Patients with proper knee balancing achieve better post-operative results, with better patient-reported outcome scores up to 6 months after surgery (5, 10). On the other hand, improper knee balancing leads to stiffness, causing instability and impaired function after TKA (9).

It has been shown that a knee can go from a balanced to an imbalanced state with simple intra-operative adjustments within only 2 degrees or 2 mm (4). Soft tissue balancing has traditionally been a subjective procedure based on the surgeon's assessment, leading to inconsistent post-surgical results (5, 10). Therefore, a quantitative, precise approach is preferred to ensure all patients have balanced knees after surgery.

TKA has a critical revision rate projected to rise by between 78% and 182% from 2014–2030 (11). A significant proportion of revision surgeries result from complications associated with improper knee balancing and instability (12). The replaced knee's instability causes excessive soft tissue laxity, leading to joint pain, effusion, and decreased function. This joint imbalance also leads to excessive MCL and Lateral Collateral Ligament (LCL) tension in varus and valgus knees, respectively. Furthermore, increased loading in these compartments leads to limitations in range of motion (4, 5).

Valgus and varus knee deformities are common knee pathologies often encountered during TKA and must be corrected to optimize surgical exposure. An integral first step for correcting either valgus or varus deformities is osteophyte removal. This increases exposure and allows the surgeon to better assess the degree of deformity intraoperatively. Various bony and soft tissue abnormalities characterize the valgus knee deformity. These include contracture of the lateral capsular and ligamentous structures and bone loss with metaphyseal remodeling (13, 14). This can pose challenges when creating balanced gaps and restoring proper limb alignment during TKA. Multiple surgical techniques have been described to balance the soft tissues in a valgus knee. Structures commonly released include the ITB, PLC, LCL, and popliteal tendon (15). Ranawat et al. proposed a technique where the PLC is palpated while the knee is held in full extension. Then, a scalpel is used to make minor cuts within the taut area until the desired correction is obtained. Though the LCL is not speciﬁcally released, many authors, including Insall and Krackow, have speculated that this technique leads to correction through the effective release of the LCL (14, 16). However, no consensus exists on best practices for creating balanced, soft tissue tension in a valgus knee.

A varus deformity usually leads to a progressive release of the medial soft tissue sleeve (MSS) by subperiosteally releasing portions of the medial soft tissue of the proximal tibia. These structures include the superﬁcial and deep MCL, semimembranosus tendon, posteromedial capsule, and the pes anserinus tendons (1, 17–19). The external MCL bundle plays a significant role in medial stability. Therefore, surgical techniques that enable progressive MCL lengthening are frequently employed (9). Correction of this deformity requires extensive medial soft tissue release during TKA (14, 17, 20, 21). Furthermore, the medial gap usually increases by approximately 20 mm after the superﬁcial MCL (sMCL) release by subperiosteally elevating its tibial insertion. In contrast, a moderate varus deformity requires only a modest (<10 mm) increase in the medial gap and may lead to over-release (22). In knees with severe medial tightness, there is a risk of complete detachment and, as a result, signiﬁcant medial instability during TKA (19). In cases of severe medial contractures, the pie-crusting technique is a valuable approach (19). Due to its precision, over-release of the medial soft tissues is prevented (19).

The pie-crusting technique is gaining prominence for balancing the knee (20). Initially, the technique was proposed for releasing the tight lateral structures in patients with mild to moderate ﬁxed valgus deformities. In early to mid-term follow-ups, the pie-crusting technique has no clinical failures or instability (4, 5, 19, 23–25). It consists of performing a series of multiple horizontal incisions or stabs in the MCL or LCL, allowing progressive lengthening adjustment while avoiding complete detachment of the ligament. Moreover, pie-crusting is a reasonably controlled technique that allows for structured soft tissue release. This is achieved by limiting the number of incisions through the ligament and controlling the amount of valgus force used to open the medial side of the knee.

This study aims to investigate the efficacy of a precise pie-crusting technique utilizing a new algorithmic grid superimposed on the LCL and MCL independently. This technique will likely allow for a more controlled release of the medial and lateral compartments.

## Methods

### Phase 1: biomechanical set-up

**Figure 1:**
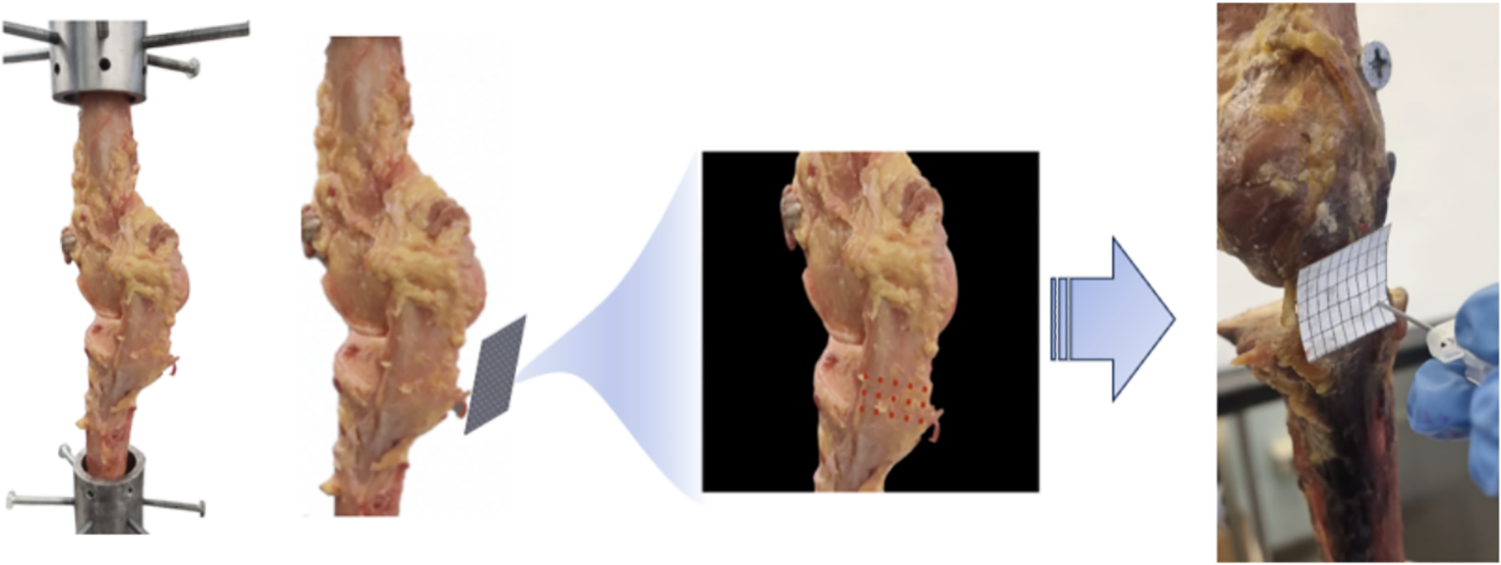
3d printed grid covering the surface of the MCL for exact perforations using an 18-gauge needle. This was in accordance with the pre-designed template/grid, as shown. The custom-made ﬁxtures were used to secure specimens in place and adequately align the knees for vertical tension.

Nine frozen cadaver knees were acquired, and all specimens were dissected, leaving only the femur and tibia connected by an isolated MCL. Similarly, 10 fresh frozen cadaver knees were dissected, leaving the femur and ﬁbula connected by an isolated LCL. In both settings, bone cuts were made similarly to those performed during primary TKA by two senior orthopedic surgery residents. Twenty-four hours prior to dissection, the specimens were wrapped in gauze, thawed at 20°C, and periodically sprayed with saline. Mechanical testing was performed using an MTS machine (Criterion Electromechanical Test System, Model C44, MTS, Minnesota, United States), fixing both the proximal segment (femur) and distal segment (tibia for MCL and fibula for LCL) of the knee into cylindrical holders. The MTS software was set to record force and displacement values following successive 18-gauge needle punctures in pairs of two at a time. This was in accordance with the pre-designed template/grid, as shown in Figure 1. The custom-made ﬁxtures were used to secure specimens in place and properly align the knees for vertical tension. Both deformations, which define the gap between the tibial plateau and femoral component transverse cut, were measured, and the linear stress-strain curve was used to compute the corresponding stiffness of the ligament (Figure 1).

### Phase 2: specimen testing

#### MCL testing

The specimens were preloaded with a controlled tensile load of 2–5 N and cycled for conditioning reasons until the desired level of stretching was attained. Previous experiments have used an 80 Newton (N) load to study the tension of the MCL (26). Our tests revealed that such load produced minimal displacement. Using the stress-strain curve, we settled on a load of 120 N due to it producing adequate displacement while the ligaments remained in the elastic range. Our testing revealed that a load exceeding 360 N was required to surpass the elastic range of the MCL load-deformation curve. We developed a patient-specific 3D-printed pie-crusting template that covers the entire surface of the ligament (Figure 1). The size of the grid was determined by measuring the medial femorotibial gap (MFT) and the width of the MCL with the knee in full extension. This grid was used as a guide during pie crusting. Horizontal outside-in perforations were made using an 18-gauge needle positioned perpendicular to the MCL ﬁbers. The holes in the grid had a diameter of 2.5 mm, allowing for smooth passage of the needle. Perforations were started in the posterior MCL at the tibial plateau level and continued in an inferior to superior and right to left direction, ultimately extending up to the transverse femoral component.

We performed 12 total perforations, four holes per row with three holes in each column, each separated by 3 mm. Measurements were performed before any perforations and after every 2 perforations for each knee. This was repeated until 12 holes were made. The resulting deformation and stiffness were recorded, and the means were computed. One specimen was severely damaged during preparation and could not be used. The results of another specimen were an outlier and were removed from the analysis.

#### LCL testing

The specimens were pre-loaded with a controlled tensile load of 2–5 N until the desired level of stretching was achieved. We opted for the 80 N load for the LCL as the specimens' width and thickness were smaller than the MCL. No further load adjustments beyond this level were performed.

An LCL grid was developed using the same techniques as described above. Due to the size and shape of the LCL, this grid included 3 holes per row and 4 per column, each separated by 3 mm.

The same testing procedure was performed for the 8 LCL knees with a total of 8 perforations made per knee. Of the 8 knees tested, one was removed due to an initial attempt to perform 12 holes, as in the case of the MCL. We found that the LCL was not large enough in caliber, both in length and width, for 12 perforations, and the experiment was continued on 8.

### Phase 3: data analysis

Data was collected after every 2 perforations. Stress–strain curve data and stiffness were calculated based on the cross-sectional area of the ligament in each specimen as determined with digital calipers. The data collected were averaged, and regression analyses were performed to determine the relationship between the number of perforations and the length and stiffness of the ligaments.

## Results

### MCL elongation

**Figure 2:**
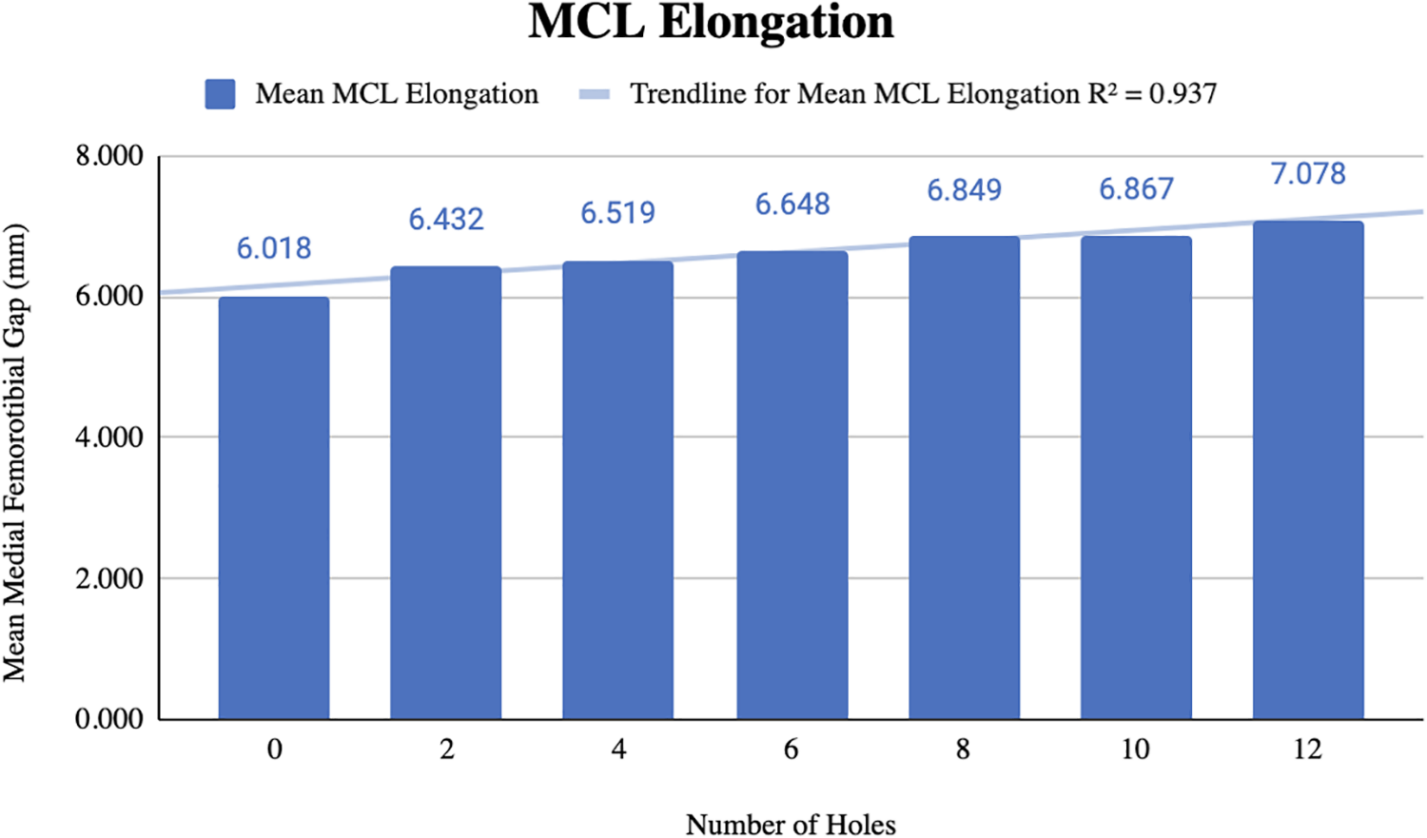
Change in MCL elongation with increasing number of perforations during pie-crusting.

Before pie-crusting, the mean medial femorotibial (MFT) space was 6.018 ± 1.4 mm (3.709–7.686 mm). After 12 perforations were made, the mean MFT increased to 7.078 ± 1.414 mm (4.975–8.680 mm) (Table 1). The mean MCL elongation was 1.060 ± 0.749 mm, with an *R*^2 ^= 0.937, indicating a strong correlation between the change in ligament length and the number of perforations (Figure 2).

**Table 1:** Medial femorotibial gap with an increasing number of perforations during pie-crusting.

	Medial femorotibial gap (mm)						
Number of Holes	0	2	4	6	8	10	12
Knee 1	7.641	7.620	7.947	7.983	7.963	8.066	8.255
Knee 2	4.973	5.060	5.194	5.220	5.179	5.281	5.462
Knee 3	3.709	3.902	4.038	4.240	4.379	4.517	4.975
Knee 5	6.432	6.601	6.906	6.944	6.843	7.060	7.235
Knee 6	7.686	7.856	8.052	8.273	9.242	8.511	8.680
Knee 7	5.412	7.259	7.499	7.499	7.788	7.939	8.059
Knee 9	6.275	6.725	5.996	6.373	6.546	6.698	6.883

### MCL stiffness

**Figure 3:**
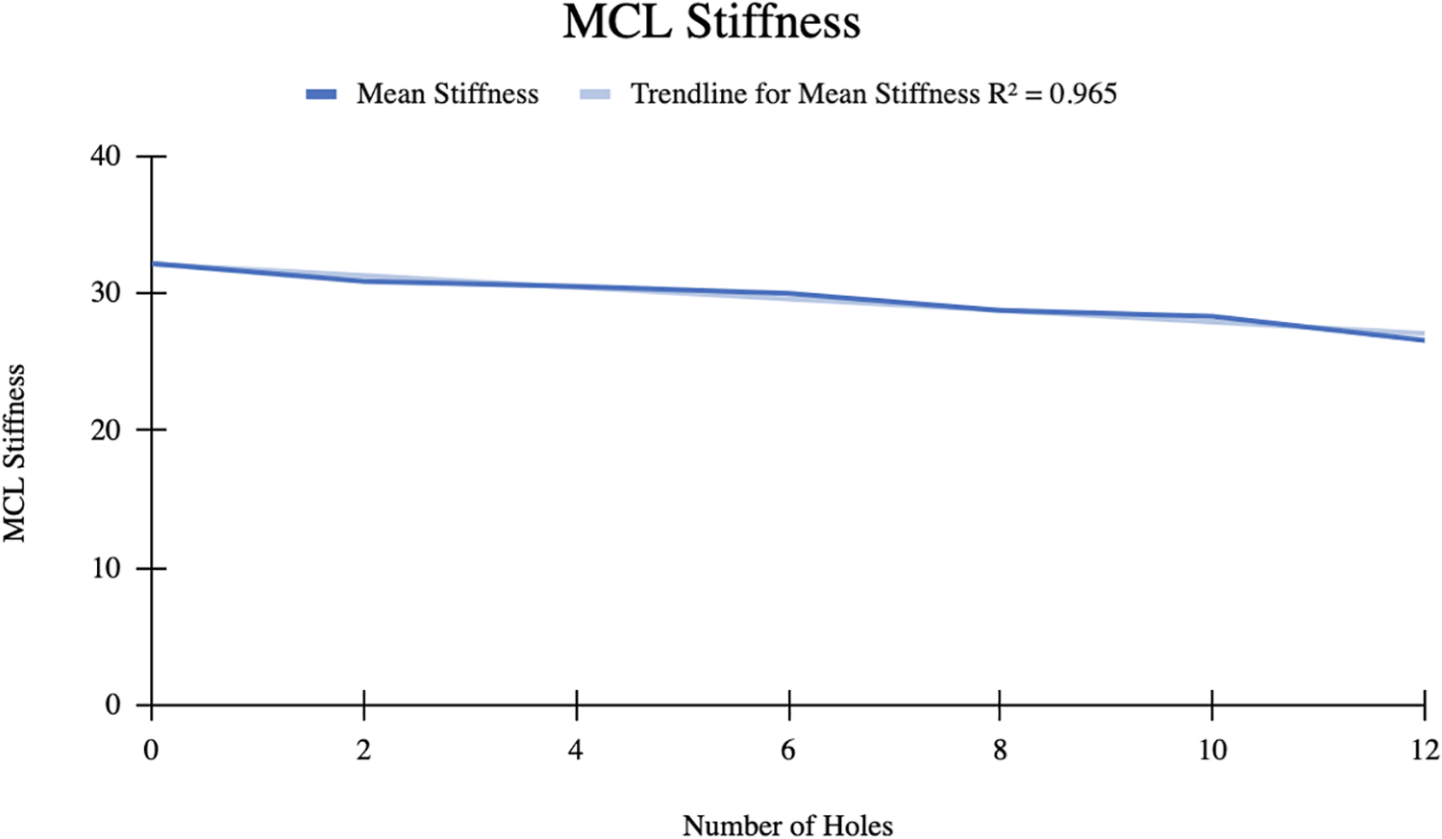
A linear relationship between mean MCL stiffness and number of perforations.

The mean MCL stiffness was 32.15 ± 9.44 N/mm initially. After 12 perforations were made, the mean stiffness decreased to 26.57 ± 6.15 N/mm (Table 2). The mean difference in MCL stiffness was 5.58 N/mm with an *R*^2^ = 0.965, indicating a strong correlation between the change in ligament stiffness and number of perforations (Figure 3).

**Table 2:** MCL stiffness with an increasing number of perforations during pie crusting.

	Stiffness (N/mm)						
Number of holes	0	2	4	6	8	10	12
Knee 1	24.71	23.57	22.96	22.46	22.36	22.46	22.27
Knee 2	35.09	34.89	34.48	33.17	31.49	31.04	29.49
Knee 3	44.9	41.13	42.46	42.42	39.73	38.83	33.8
Knee 5	22.38	22.6	21.42	21.32	23.85	22.82	22.23
Knee 6	22.55	22.59	22.38	22.6	19.14	20.39	18.9
Knee 7	43.17	41.25	39.31	37.81	37.22	35.95	34.69
Knee 9	32.27	30.08	30.46	30.18	27.41	26.61	24.61

### LCL elongation

**Figure 4:**
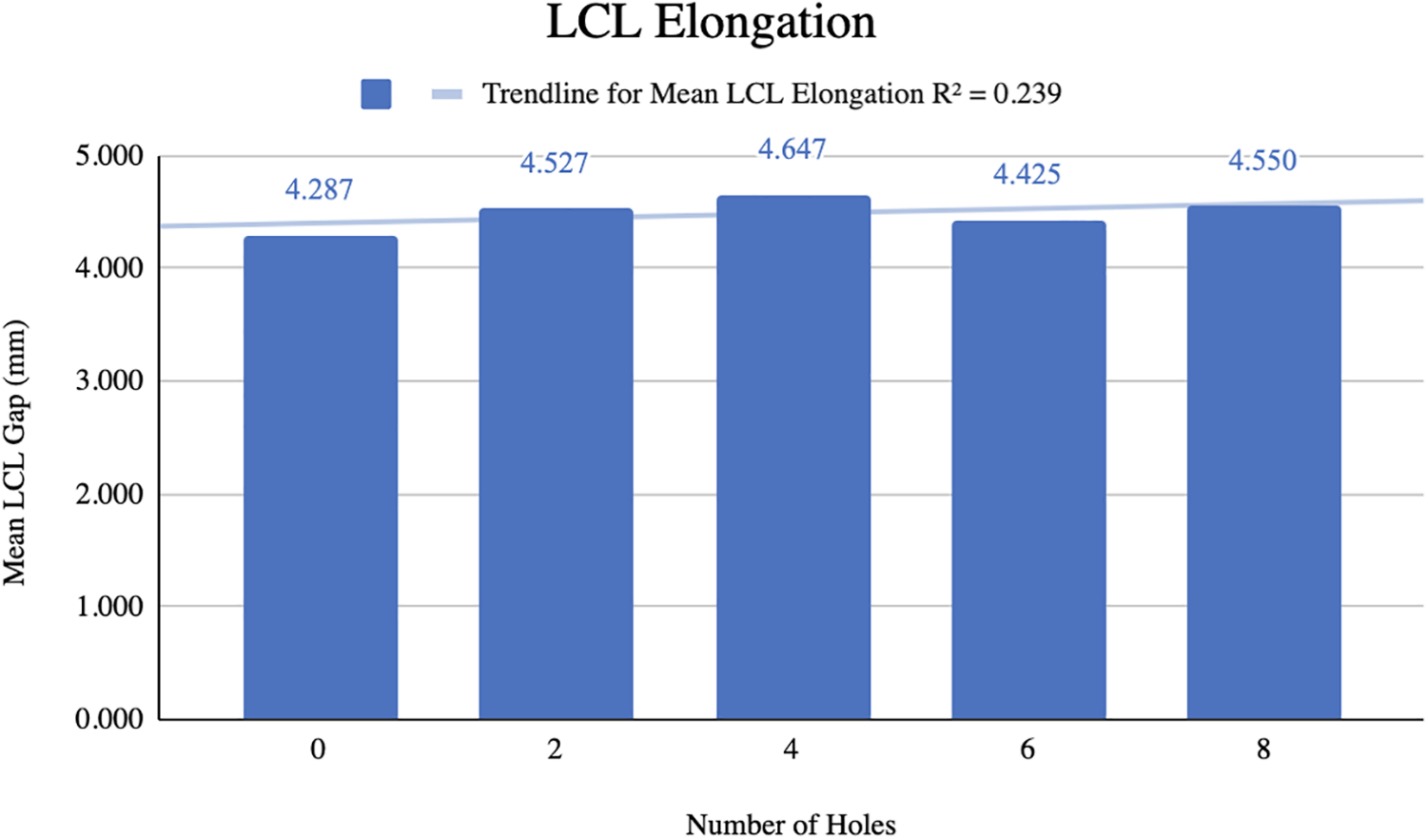
Change in mean LCL gap with the progression of pie-crusting.

The mean gap between the femur and ﬁbula before pie-crusting was 4.287 ± 0.911 mm (Figure 4). After 8 perforations were made, the mean gap increased to 4.550 ± 0.876 mm resulting in a mean difference of 0.263 mm. The mean LCL gap generally increased as the number of perforations increased, although this relationship was relatively weak (*R*^2^ = 0.239).

### LCL stiffness

**Figure 5:**
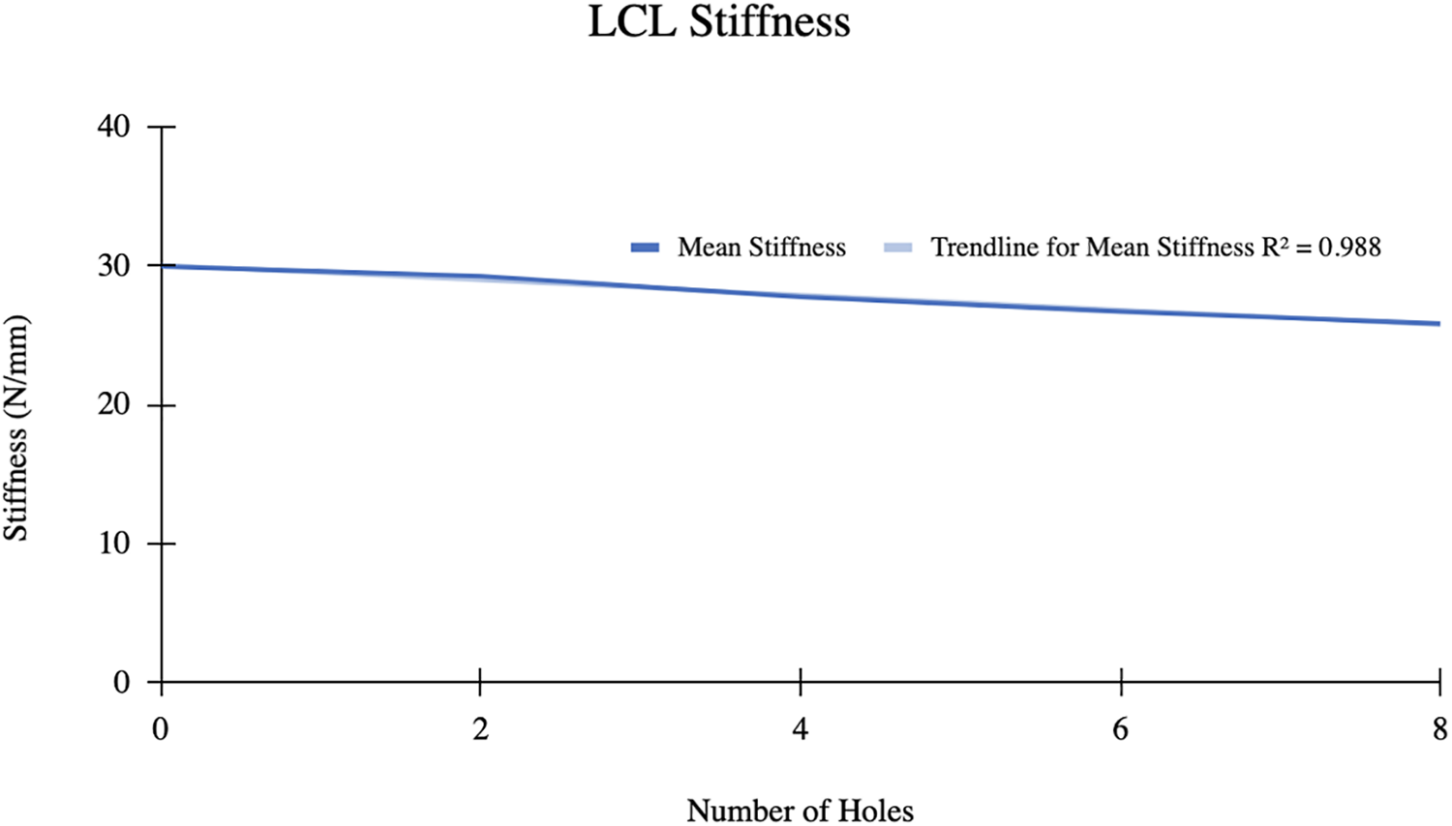
A linear relationship between mean LCL stiffness and number of perforations.

The mean LCL stiffness before pie crusting was 29.96 ± 5.24 N/mm (Figure 5). After 8 perforations, the mean stiffness decreased to 25.85 ± 6.31 N/mm. Mean LCL stiffness decreased by 4.11 N/mm and displayed a strong inverse relationship with the number of perforations (*R*^2^ = 0.988).

## Discussion

In the MCL group, the 7 knees tested showed a statistically significant linear relationship whereby MFT space and MCL elongation increase as a function of increasing punctures made by the 18-gauge needle. The mean increase in MFT space was 7.078 ± 1.414 mm after 12 punctures, with the pie-crusting technique providing 1.060 mm MCL elongation, on average. Additionally, there was a statistically significant reduction in MCL stiffness as a function of increasing punctures. The mean MCL stiffness after 12 punctures was 26.57 ± 6.15 N/mm, a reduction of 5.58 N/mm from the initial MCL stiffness. Similar to the MCL group, knees in the LCL group showed a linear relationship whereby femur-fibula gap and LCL elongation increase as a function of increasing punctures made by the 18-gauge needle. Following 8 perforations, the mean length of the LCL increased from 4.287 ± 0.911 mm–4.550 ± 0.876 mm. With an increasing number of perforations performed, the stiffness of the LCL significantly decreased without sacrificing the integrity of the ligament. These results suggest that utilizing this grid for pie-crusting allows for gradual lengthening and release of both the MCL and LCL.

Varus and valgus knee deformities present challenges for restoring proper alignment and adequate balance. Osteophyte removal is often a key component of alignment restoration in TKA. However, osteophytes are mostly found on the distal femur and proximal tibia which will be effectively removed once bone cuts are made. Therefore, soft tissue release is necessary when deformities remain following bone cuts. Extensive soft-tissue releases are associated with significant complications. Likewise, traditional soft tissue balancing during TKA is determined by the surgeon's subjective assessment. Together, these obstacles and inexact methodologies contribute to nearly 22% of the revision of total knee arthroplasties being performed (27).

In cases of varus deformity, achieving balance requires a gradual release of the medial soft tissue sleeve (MSS) from the proximal medial tibia. The well-known medial soft tissue release method, introduced by John Insall et al., involves precise subperiosteal dissection from the proximal medial tibia. This dissection encompasses both superficial and deep medial collateral ligaments (MCL), the semimembranosus tendon, the posteromedial capsule, and, optionally, the pes anserinus tendons (1). Nonetheless, this technique needs to improve in selectively addressing flexion and extension gap releases. As such, this investigation aims to present a refinement of the pie-crusting technique to address varus and valgus deformity correction in TKA patients.

While prior studies have posited that pie-crusting may only be successful for 2–4 mm of medial tightness (4), here we show that MFT spaces ranging from 3.7 mm–7.6 mm can be lengthened using the pie-crusting technique. Kwak et al. previously reported that pie-crusting using a blade knife is unreliable for correcting varus deformities with MCL release (28). Whereas they show the unexpected early over-release of the MCL, leading to joint instability, our technique using an 18-gauge needle did not lead to over-release or mechanical instability. Moreover, Kwak et al. report that blade pie-crusting led to 70% over-release, mainly when performed in the flexed knee (28). Our results concur with previous work published by Koh et al., which showed that needle puncture pie-crusting produced more consistent increases in the medial gap than blade pie-crusting (5). Therefore, with regards to increasing MCL elongation and reducing stiffness, using a novel grid-based approach to pie-crusting provides an expedient, effective, safe, and beneficial technique for addressing varus deformity in TKA patients.

While TKA's are standard for addressing valgus deformities of the knee, there is a lack of consensus on optimal soft tissue release techniques. Multiple lateral soft tissue release techniques exist in the literature (29). Krackow utilized an early LCL release with subsequent popliteus tendon and ITB release to grade the release of the lateral structures. This resulted in a uniform release of the joint gap but tended toward external rotation of the tibia (16). Whiteside tested a selective stepwise ligament release approach. The LCL and popliteus tendon were released first, followed by the ITB and then the PLC only if the knee remained significantly tight in extension. Following the ligamentous release, patients had improved knee alignment with no evidence of clinical instability (30). Boettner et al. proposed a “cook-book” approach to soft tissue release that could be broadly used for all patients with valgus deformity. It begins with ITB release at the joint line level, which is then extended laterally to release the LCL and PLC while preserving the popliteal tendon. Patients undergoing this release significantly improved knee flexion and alignment without increased complications (31). Xie et al. performed a lateral retinacular release (LRR) during TKA for valgus-deformed knees (29). This technique led to improvements in knee alignment without increasing instability or needing revision. Xie concluded that a selective soft tissue release is optimal compared to the standard “cookbook” approach. However, no comparative studies exist between the various release techniques.

Prior biomechanical studies have shown that pie-crusting without effective release of the LCL leads to only a small amount of valgus correction (32). However, Aglietti et al. showed that selective pie-crusting of the lateral structures, including the PLC, LCL, and ITB, during TKA led to adequate correction of valgus misalignment while reducing the risk of posterolateral instability. Our study sought to examine an algorithmic approach to soft tissue release through pie-crusting of only the LCL for valgus knee deformity. Although no studies have specifically examined the effects of pie-crusting only the LCL during TKA, our findings indicate that algorithmic LCL pie-crusting decreases lateral soft tissue tightness. Thus, LCL pie-crusting may be utilized as part of a selective soft tissue release protocol during TKA for valgus-deformed knees. Additionally, percutaneous LCL pie-crusting has been shown to increase access and working space of the lateral knee during arthroscopy (33); therefore, using the proposed grid to perform stepwise LCL loosening may also be advantageous during various knee arthroscopy procedures.

The introduction of robotic platforms has enhanced the customization of alignment in knee arthroplasty, tailoring it to individual bony anatomy and native ligament balancing (34). Some robotic-assisted platforms incorporate 3D pre-operative planning, utilizing imaging modalities such as CT scans, to assess bony anatomy in all three planes. A benefit of 3D-imaging is the assessment of the axial plane, providing a more intricate preoperative analysis of femoral and tibial rotation. This allows for optimal implant positioning and sizing (34). These systems enable a quantifiable intraoperative assessment of soft tissue balancing along with the ability to make precise adjustments leading to restoration of joint line height and obliquity (35). Preliminary studies using such robotic-assisted systems have demonstrated favorable gap balancing, restoration of native knee alignment, including partial correction of varus deformity, and improved post-operative functioning compared to mechanical alignment techniques (36–38). Furthermore, these techniques lead to less soft tissue damage compared to conventional manual techniques (39). However, these systems are costly and take time to become comfortable with. Thus, more effective manual strategies for soft tissue balancing will prove beneficial to surgeons without access to this technology.

For surgeons with access to assistive technologies, the pie-crusting technique could be seamlessly integrated alongside manual and robotic tensioner devices. Tensioners allow the surgeon to quantify ligament tension as well as medial and lateral gaps in real-time (40). Rossi et al. demonstrated promising clinical results at mid-term follow-up using a manual tensioner device during TKA (41). As part of their surgical protocol, if a mismatch of greater than 2–3 degrees (or mm) was measured between the medial and lateral compartments, then ligamentous release via needle crusting was performed. Thus, the more precise pie-crusting technique described in our study could also be used alongside tensioner devices and may lead to superior outcomes. After each pair of perforations, the surgeon would be confident in the degree of soft tissue release achieved and how much more would be needed to correct a varus or valgus misalignment.

This study has several limitations. First, only 7 knees were included in the MCL group, and only 6 knees were included in the LCL group for data analysis. Although this is a small sample size compared to clinical trials, small samples are relatively common in biomechanical studies. Our study also utilized cadaveric knees with unknown pre-existing alignment. Thus, they may not be an accurate model of the asymmetric soft tissue tightness seen in valgus and varus-deformed knees. This makes it difficult to discern the effectiveness of MCL and LCL pie-crusting in patients with true varus and valgus deformities, respectively. The age and sex of the cadavers were also not accounted for in our analysis, as this may have affected the relative stiffness of the ligaments. Additionally, the dynamic stabilizers of the knee were not intact during testing. These include the ITB, popliteus tendon, and lateral head of the gastrocnemius. Ultimately, this decreases the generalizability of our results to patients with intact soft tissue structures surrounding the knee. Performing the pie-crusting solely on the MCL or LCL also significantly differs from current techniques for soft tissue release during TKA. Furthermore, all the testing was performed with knees in an extended position, making it challenging to assess the effects of pie-crusting when the knee is flexed.

The soft tissue balance is fundamentally crucial in total knee arthroplasty. When bone cuts fall short in restoring coronal balance, the collateral ligaments play a pivotal role. They can restore coronal balance in varus and valgus knees with tight medial and lateral gaps, respectively, aiding in maintaining the screw home mechanism—an essential aspect of sagittal balance. This study demonstrates the safety and efficacy of our algorithmic pie-crusting technique of the MCL and LCL employing a customized grid. This pie-crusting technique can reassure controlled gap balancing without compromising knee stability, achieving both coronal and sagittal balance. The technique also avoids specific complications such as over-release or mechanical instability. Prior instances of medial release using pie-crusting encountered issues of over-release, a notable concern. We attribute these complications to blade pie-crusting and imprecise punctures. In contrast, our approach mitigates both concerns by utilizing an 18-gauge needle and a pre-formed grid. The inherent safety, simplicity, and high success rate of the needle pie-crusting technique validate its routine application for addressing varus and valgus deformities. Ultimately, future studies should examine the effects of an increased number of holes through this pie-crusting technique to evaluate the number needed for optimal soft tissue release.

## Data Availability

The raw data supporting the conclusions of this article will be made available by the authors, without undue reservation.
